# Varicella Zoster Virus in the Nervous System

**DOI:** 10.12688/f1000research.7153.1

**Published:** 2015-11-26

**Authors:** Don Gilden, Maria Nagel, Randall Cohrs, Ravi Mahalingam, Nicholas Baird

**Affiliations:** 1 Department of Neurology, University of Colorado School of Medicine, Aurora, Colorado, 12700, USA; 2 Department of Immunology & Microbiology, University of Colorado School of Medicine, Aurora, Colorado, 12800, USA

**Keywords:** Varicella zoster virus, VZV, Latency, Vasculopathy, Giant Cell Arteritis, Immunization

## Abstract

Varicella zoster virus (VZV) is a ubiquitous, exclusively human alphaherpesvirus. Primary infection usually results in varicella (chickenpox), after which VZV becomes latent in ganglionic neurons along the entire neuraxis. As VZV-specific cell-mediated immunity declines in elderly and immunocompromised individuals, VZV reactivates and causes herpes zoster (shingles), frequently complicated by postherpetic neuralgia. VZV reactivation also produces multiple serious neurological and ocular diseases, such as cranial nerve palsies, meningoencephalitis, myelopathy, and VZV vasculopathy, including giant cell arteritis, with or without associated rash. Herein, we review the clinical, laboratory, imaging, and pathological features of neurological complications of VZV reactivation as well as diagnostic tests to verify VZV infection of the nervous system. Updates on the physical state of VZV DNA and viral gene expression in latently infected ganglia, neuronal, and primate models to study varicella pathogenesis and immunity are presented along with innovations in the immunization of elderly individuals to prevent VZV reactivation.

## Introduction

Varicella zoster virus (VZV) is an exclusively human alphaherpesvirus. Primary infection causes varicella (chickenpox), after which the virus becomes latent in neurons of cranial nerve ganglia, dorsal root ganglia, and autonomic ganglia along the entire neuraxis. In elderly and immunocompromised individuals, a decline in VZV-specific cell-mediated immunity results in VZV reactivation, typically manifesting as herpes zoster (shingles) in a dermatomal distribution. The annual medical care cost of treating approximately one million cases of zoster in the US is estimated at $1.1 billion, most of which is used to treat immunocompetent adults who are at least 50 years old ^1^. Since the risk of zoster increases with age and since the population that is at least 65 years old is expected to increase to 72 million in 2030, zoster and its attendant serious neurological complications will continue to be a significant health-care burden.

Zoster is often complicated by chronic pain (postherpetic neuralgia). VZV reactivation can also produce cranial nerve palsies, meningoencephalitis, VZV vasculopathy, myelopathy, and ocular disease with or without associated rash. Recently, the spectrum of VZV vasculopathy has expanded to include giant cell arteritis (GCA), which is the most common systemic vasculitis in the elderly and which frequently results in vision loss. Herein, we review the clinical, laboratory, imaging, and pathological features of neurological complications of VZV reactivation as well as diagnostic tests to verify VZV infection of the nervous system. We also discuss VZV latency, neuronal and primate models to study varicella pathogenesis and immunity, and immunization of elderly individuals to prevent VZV reactivation.

## Neurological complications of VZV reactivation

### Herpes zoster

Most cases of VZV reactivation manifest as zoster (dermatomal distribution pain and rash). Rash and pain usually occur within hours to days of each other, although pain may precede rash by weeks to months ^2^. Since VZV is latent in ganglia along the entire neuraxis, zoster can develop anywhere on the body. Risk factors for the development of zoster include advanced age ^3^ and immunosuppression, such as in individuals with AIDS or cancer, organ transplant recipients, and individuals who received immunosuppressive medications ^4^. Zoster in an otherwise healthy young person may be the first manifestation of HIV infection ^5^; zoster in children or adolescents is also seen in individuals who acquired primary VZV infection *in utero* or in the first year of life; these people are 20.9 times more likely to develop zoster before age 20 ^6^.

Since VZV is latent in all cranial nerve ganglia and in autonomic (ciliary and otic) ganglia in the head, multiple cranial nerve palsies can develop after zoster. Ophthalmoplegia from involvement of cranial nerve 3, 4, or 6 or any combination thereof is a well-documented complication. Another is weakness or paralysis of facial muscles on one side of the face, associated with vesicles in the ipsilateral external auditory canal (zoster oticus), or on the tympanic membrane, the ipsilateral anterior two thirds of the tongue, or hard palate. The combination of peripheral facial palsy and zoster oticus constitutes the Ramsay Hunt syndrome (RHS). Because the facial nerve is adjacent to the eighth cranial nerve in the facial canal, patients with RHS often have tinnitus, hearing loss, nausea, vomiting, vertigo, or nystagmus. Cranial neuropathies usually occur days to weeks after zoster. The temporal relationship most likely reflects the time needed for the virus to spread transaxonally along trigeminal and other ganglionic afferent fibers, thereafter replicating in small arteries with resultant micro-infarction of cranial nerves (as occurs in patients with diabetes) in the same manner that produces VZV vasculopathy in larger arteries. Importantly, cranial neuropathies produced by VZV can occur in the absence of rash, virologically verified by the detection of VZV DNA or anti-VZV IgG antibody in cerebrospinal fluid (CSF).

Zoster paresis is characterized by weakness in the arm or diaphragm after cervical distribution zoster or in the leg after lumbar or sacral distribution zoster. Thoracic zoster has been associated with abdominal muscle weakness and hernia. Pathological features of zoster include inflammation and hemorrhagic necrosis with associated neuritis, localized leptomeningitis, unilateral segmental poliomyelitis, and degeneration of related motor and sensory roots ^7^. Demyelination may be seen in areas with mononuclear cell (MNC) infiltration and microglial proliferation. In acutely infected ganglia, intranuclear inclusions, viral antigen, and herpesvirus particles are present. Oral antiviral drugs speed healing of zoster rash and shorten the duration of acute pain. Immunocompromised patients and patients with ophthalmic-distribution zoster should receive intravenous acyclovir (10 to 15 mg/kg three times daily for 5 to 7 days).

## Postherpetic neuralgia

Postherpetic neuralgia (PHN) is defined as pain persisting for more than 3 months after zoster. Age is the single most important predictor, and more than 40% of zoster patients who are more than 60 years old develop PHN. Pathological analysis of ganglia from an early case of PHN of 2.5 months’ duration revealed diffuse and focal infiltration by chronic inflammatory cells, a finding confirmed by the detection of prominent collections of lymphocytes in ganglia from a patient with PHN of 2 years’ duration ^8^. A possible explanation is that chronic inflammation reflects prolonged viral infection, a notion supported by the detection of VZV DNA in blood MNCs of many patients with PHN (presumably by MNCs trafficking through ganglia productively infected with VZV) and from the favorable response of some patients with PHN to antiviral treatment ^9^. Symptomatic treatment for PHN is challenging. Tricyclic antidepressants, gabapentin, and pregabalin are used as first-line therapies. Many patients with PHN also require topical capsaicin cream, lidocaine patches, capsaicin 8% patches, tramadol, or opioids or a combination of these to help alleviate debilitating pain. Nerve blocks and ablation, as well as nerve stimulators, have variable effectiveness. The potential role of antiviral medications as a treatment for PHN awaits analyses in larger clinical studies.

## Central nervous system disease caused by VZV reactivation

VZV meningitis, meningoencephalitis, meningoradiculitis, cerebellitis, myelopathy, and vasculopathy may develop after zoster. Importantly, all may develop in the absence of rash, as confirmed by the detection of VZV DNA or anti-VZV antibody (or both) in CSF. VZV myelitis commonly presents as frank invasion of the spinal cord by the virus. Disease is usually progressive and infrequently fatal. Magnetic resonance imaging reveals longitudinal serpiginous-enhancing lesions ^10^. Early diagnosis and aggressive treatment with intravenous acyclovir are beneficial, even in immunocompromised patients ^11^; VZV myelitis may recur, even in immunocompetent patients ^10^. Aside from myelitis, VZV can produce spinal cord infarction from occlusion of spinal arteries ^12^.

VZV vasculopathy occurs following productive infection of cerebral arteries and pathological vascular remodeling. The most common manifestations are ischemic stroke, but hemorrhagic stroke, aneurysm, with and without subarachnoid and intracerebral hemorrhage, dolichoectasia, dissection, and venous sinus thrombosis may also be produced by VZV. Although the exact incidence of VZV vasculopathy is unknown, epidemiological studies from Taiwan, Denmark, and the UK have all revealed that the risk of stroke is increased after zoster, particularly when zoster is in the ophthalmic distribution of the trigeminal nerve ^13^, and that antiviral therapy may reduce stroke risk. Retrospective analysis of 30 subjects with VZV vasculopathy, confirmed virologically, revealed that 63% had associated rash, 67% had a CSF pleocytosis, 97% had abnormalities on brain imaging, and 70% had abnormalities on angiographic studies ^14^. Of the 30 subjects, 50% had both large and small artery involvement, 37% had exclusively small artery involvement, and 13% had exclusively large artery involvement. Because 93% of the subjects had intrathecal synthesis of anti-VZV antibodies and only 30% had VZV DNA in CSF, the detection of anti-VZV IgG antibody is the best test for diagnosis consistent with a prior study ^15^. Importantly, since there was no zoster rash in one third of subjects, VZV DNA is absent in 70% of CSF, and there is a 4.2-month delay from onset of rash to neurological disease, thus the diagnosis of VZV vasculopathy is often missed.

The proposed mechanism for VZV vasculopathy involved virus reactivation from cranial nerve ganglia followed by transaxonal spread of the virus to the outermost adventitial layer of cerebral arteries whereupon productive virus infection induces pathological vascular remodeling through a direct effect or an indirect effect of virus-induced inflammation. Immunohistochemical studies of VZV-infected cerebral and temporal arteries from patients with VZV vasculopathy show that arteries contain a thickened intima composed of myofibroblasts, loss of medial smooth muscle cells, and a disrupted/duplicated internal elastic lamina ^16^. Examination of the inflammatory infiltrate in VZV-infected arteries reveals CD4 and CD8 T cells, macrophages, and rare B cells predominantly located in the adventitia and to a lesser extent in the thickened intima ^17^. In early VZV vasculopathy, neutrophils are present in the adventitia; no neutrophils were detected in late VZV. Consistent with studies of pathological remodeling in coronary and pulmonary arteries, the thickened intima was associated with inflammation in the underlying adventitia, supporting the notion that inflammatory cells secrete soluble factors that contribute to vascular remodeling. A recent study also revealed that VZV-infected brain vascular adventitial fibroblasts produce elevated levels of matrix metalloproteinases that degrade extracellular matrix and may contribute to migration of cells to the lumen and thrombosis as well as to aneurysm formation and hemorrhage ^18^.

## Association of VZV with giant cell arteritis

**Figure 1.**
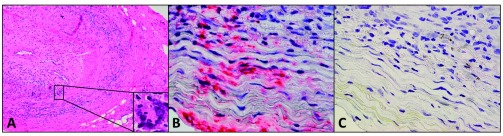
Detection of varicella zoster virus (VZV) antigen in the temporal artery of a patient with giant cell arteritis. Hematoxylin-and-eosin staining of the temporal artery from a 90-year-old man with classic giant cell arteritis ( **A**). Note extensive inflammation in the adventitia, media and intima, disruption of the media, a thickened intima, and nearly occluded arterial lumen; inset denotes a giant cell. Immunohistochemical staining with mouse anti-VZV gE antibody revealed VZV antigen (red) in the adventitia and media of the temporal artery ( **B**) that was not seen when mouse isotype IgG1 antibody was substituted for mouse anti-VZV gE antibody ( **C**).

Evidence that VZV infection triggers the inflammatory cascade characteristic of GCA came from analysis of formalin-fixed, paraffin-embedded GCA-positive temporal artery (TA) biopsies and normal TA biopsies from subjects who are more than 50 years of age for the presence and distribution of VZV antigen. VZV antigen was found in 61 out of 82 (74%) GCA-positive TAs compared with 1 out of 13 (8%) normal TAs, and most GCA-positive TAs contained viral antigen in skip areas ^19^. VZV antigen was present mostly in adventitia, followed by the media and intima ( Figure 1). Hematoxylin-and-eosin staining revealed VZV antigen in 12 out of 32 (38%) skeletal muscles adjacent to VZV antigen-positive TAs. Despite formalin fixation, polymerase chain reaction (PCR) detected VZV DNA in 18 out of 45 (40%) GCA-positive VZV antigen-positive TAs, in 6 out of 10 (60%) VZV antigen-positive skeletal muscles, and in one VZV antigen-positive normal TA. Electron microscopy revealed VZV virions in a GCA-positive TA. GCA pathology in sections adjacent to those containing VZV was seen in 89% of GCA-positive TAs but in none of 18 adjacent sections from normal TA. Most GCA-positive TAs contained VZV in skip areas that correlated with adjacent GCA pathology, supporting the notion that VZV triggers GCA immunopathology.

The association between GCA and TA infection by VZV was further analyzed by immunohistochemistry in archived TAs of patients with clinically suspected GCA whose TAs were negative histopathologically, and in normal TAs removed postmortem from age-matched subjects ^20^. VZV antigen was detected in 45 out of 70 (64%) GCA-negative TAs compared with 11 out of 49 (22%) normal TAs, and extension of our earlier study revealed VZV antigen in 68 out of 93 (73%) GCA-positive TAs compared with 11 out of 49 (22%) normal TAs. VZV antigen was more likely to be present in adventitia of both GCA-negative TAs and GCA-positive TAs than in normal TA adventitia. In GCA-negative subjects whose TAs contained VZV antigen, adventitial inflammation was seen in 36% adjacent to viral antigen but not in any normal TAs. Overall, the prevalence of VZV in TAs of subjects with clinically suspected GCA was independent of whether they were GCA-negative or -positive pathologically.

## VZV latency

During primary infection, VZV disseminates hematogenously and infects ganglionic neurons along the entire neuraxis. Neurons may also become infected by retrograde axonal transport of the virus from skin vesicles. In non-neuronal cells, VZV replicates and causes cell death, whereas VZV does not replicate in or kill neurons; rather, transcription of virtually all viral genes is silenced ^21^. Serologic evidence shows that more than 95% of the world’s population is exposed to VZV ^22^, and most autopsy studies have found latent VZV DNA in trigeminal ganglia from more than 87% of individuals ^23^. Whereas latent VZV is innocuous, reactivation results in significant disease (see above). Thus, understanding molecular aspects of virus latency is necessary to design therapeutic interventions to inhibit virus reactivation.

In latently infected human trigeminal ganglia, one to five copies of VZV DNA are present in 1.5% of the approximately 27,000 neurons ^24^. Whereas VZV double-stranded DNA is linear in infectious virus particles, VZV DNA is ‘endless’, most likely existing as circular episomes in the nucleus of latently infected neurons ^25^. VZV encodes about 70 genes, all of which are transcribed during productive infection ^26^, but in latently infected human ganglia, only VZV gene 63 RNA is found in the first 9 hours after death ^27^ and fewer than 12 VZV genes are transcribed in the next 15 hours ^28, 29^. Regulation of VZV genes during latency, an active area of research, most likely involves post-translational modification of histone proteins that comprise nucleosomes bound to promoters of virus genes. Nucleosomes containing histone protein 3 (H3) post-transcriptionally modified by acetylation on lysine 9 (K9ac) are present on promoters of actively transcribed cell genes ^30^. Analysis of VZV DNA in human trigeminal ganglia shows that H3K9ac-containing nucleosomes are also present on the promoters of actively transcribed virus genes (VZV genes 21 and 63) but not on virus genes that are transcriptionally silent (VZV genes 14 and 36) ^31^, findings which suggest that latent VZV gene transcription is, in part, regulated epigenetically. Thus, novel therapies that inhibit specific histone modifications may reduce virus reactivation ^32^. Although the initial steps in virus reactivation may involve histone modifications on the latent virus that increase the number of VZV genes transcribed ^27^, the end result of virus reactivation is assembly and release of infectious virions, with extensive cell death and induction of major histocompatibility complex (MHC) class I and II protein with CD4 and CD8 T-cell recruitment ^33– 35^.

Because VZV is an exclusively human pathogen, studies of VZV latency have been limited to analysis of human ganglia obtained postmortem. However, molecular analysis of latently infected primate ganglia with simian varicella virus (SVV) has provided useful information about varicella latency, and recent *in vitro* studies of neurons experimentally infected with VZV offer promise to study mechanisms of VZV reactivation (see below). The immediate future of research on VZV latency will involve correlative analyses of latently infected human ganglia, primate ganglia latently infected with SVV, and *in vitro* models of neurons experimentally infected with VZV, which can be exploited to prevent serious neurological, ocular, and visceral diseases produced by VZV reactivation.

## Efforts to produce VZV latency and reactivation in animals

Animal models developed for VZV latency using rodents and primates do not fulfill the criteria for VZV latency, which include detection of VZV DNA exclusively in ganglia, detection of latent VZV only in neurons, restricted transcription of the virus genome, and ability to reactivate the virus. Inoculation of VZV into guinea pigs ^36^, mice ^37^, and rats ^38^ resulted only in seroconversion. In intradermally inoculated guinea pigs, VZV nucleic acids and proteins were still found in enteric ganglia, stomach, ileum, and colon of guinea pigs 1 month later ^39^. In non-human primates, experimental inoculation of VZV produced a humoral and cell-mediated immune response, but the virus did not become latent in ganglia ^40^.

However, clinical, virological, immunological, and pathological features of SVV infection in non-human primates closely parallel VZV infection in humans ^41, 42^, and SVV becomes latent exclusively in ganglionic neurons ^43, 44^. SVV-specific transcription (open reading frames 21, 62, 63, and 66) is limited in ganglia, and the virus can be reactivated by immunosuppression ^45^. SVV antigens are present in macrophages and dendritic cells during reactivation ^46^. Primate models of varicella pathogenesis have revealed that the virus enters ganglia hematogenously before the appearance of skin rash ^47^, memory T cells disseminate SVV to lung and ganglia during primary infection ^48^, and T-cell infiltration correlates with expression of CXCL10, a chemokine that recruits activated T cells and natural killer (NK) cells, in ganglia at the time of zoster ^49^. Pro-inflammatory cytokines and chemokines and anti-inflammatory mediators are elevated at the time of zoster in immunosuppressed monkeys ^50^. All T-cell subsets decreased during immunosuppression and peaked (except for CD8 T cells) 2 weeks before zoster ^51^.

## Neuronal models for VZV latency

In latently infected human ganglia, the entire VZV genome is present with termini that are covalently linked (i.e., either circular or concatemeric (end-to-end)) ^25^, viral transcription is severely restricted ^27, 29, 52, 53^, and the virus can reactivate. An *in vitro* model of VZV latency that satisfies all three of these conditions has not yet been developed.

Neuroblastoma cell lines, including SH-SY5Y, are malignant cell lines of neurogenic origin that can be induced to differentiate *in vitro* to a neuron-like cell ^54^. However, differentiation into neurons is inefficient (only approximately 70% are differentiated as determined by phase-contrast microscopy) and infection of the remaining non-neuronal cells leads to productive infection.

Induced pluripotent stem cells (iPSCs) are fibroblasts that have been de-differentiated to a stem cell-like state and then induced to differentiate into neurons. Infection of iPSCs with a low dose of VZV results in a slowly spreading productive infection that produces viral DNA, transcripts, protein, and infectious virus ^55, 56^. A cytopathic effect does not develop until about 4 weeks after infection ^57^, whereas similarly infected fibroblasts die in 7 to 10 days. Although this is promising, additional studies are necessary to suppress viral DNA replication, transcription, and virion production to mimic VZV latency *in vivo*.

Two other human stem cells have also been used to study the VZV-neuron relationship *in vitro*: neural stem cells (hNSCs) and embryonic stem cells (hESCs). At 9 weeks’ gestation, hNSCs were purified from fetal brain, resulting in approximately 90% neuron purity as indicated by staining for the neuronal markers MAP2a and β-tubulin ^58^. Two weeks after infection with low-dose VZV, no cytopathic effect was evident, although viral DNA, transcripts, and protein were all detectable. hESCs are derived from the inner cell mass of *in vitro* fertilized human embryos and can be maintained undifferentiated for at least 8 months in culture ^59^. After differentiation *in vitro*, VZV infection of hESC-derived neurons results in a lytic infection ^60, 61^. Recently, Markus and colleagues ^62^ claimed to have developed an *in vitro* model of latency with reactivation of VZV. Although the data in their report do not recapitulate what is known of VZV latency in human ganglia (see above), their model does advance the VZV field since it represents long-term (over a 7-week period), low-level infection, which can be stimulated with various pharmacological agents to boost viral biogenesis (DNA replication, transcription, and translation).

Normal human neural progenitor cells can be grown on inert microbeads inside NASA-designed rotary vessels to form tissue-like assemblies (TLAs) that resemble the structure of human ganglia obtained at autopsy ^63^. Cells in the TLA express both progenitor and mature neuronal markers and can be maintained for at least 6 months. VZV infection of TLAs results in virus transcription and DNA replication during the initial approximately 18 days of infection, after which a steady-state characterized by persistent infection with sporadic release of virus is reached.

Overall, the iPSCs, hESCs, and TLA cells have provided prolonged VZV infection of neurons before lytic infection develops but have not produced VZV latency. Further studies involving the use of cytokines, antivirals, and other compounds are needed to possibly achieve latency *in vitro*.

## Vaccination to prevent varicella and zoster

### Varivax

In 1995, varicella vaccine became available in the US. The Centers for Disease Control and Prevention (CDC) recommends immunization at 1 year of age and a second dose at age 4 to 6. Contraindications to the vaccine include life-threatening allergy to previous administration, gelatin or neomycin, pregnancy, and severe immunosuppression. Among states that accurately reported the incidence of varicella, disease declined by 79% overall between 2000 and 2010 and this was likely due to vaccine.

### Zostavax

In 2005, the Shingles Prevention Study, a randomized placebo-controlled trial, provided evidence that the herpes zoster vaccine reduced the incidence of zoster and PHN in the elderly ^64^. In that study, 38,546 healthy adults at least 60 years of age (median of 69 years) were randomly assigned to receive a single dose of attenuated VZV with 14-fold higher potency than varicella vaccine or placebo. The higher-potency vaccine was required to increase VZV-specific cell-mediated immunity in latently infected older adults. The incidence of zoster as well as the burden of illness due to zoster (total pain and discomfort) and the incidence of PHN were determined. Average follow-up of 3.13 years in a total of 19,270 people who received zoster vaccine and 19,276 who received placebo revealed 957 confirmed cases of zoster (315 in vaccine recipients and 642 in placebo recipients). In both groups, more than 93% of the subjects with zoster were positive for wild-type VZV DNA by PCR; none had vOka DNA. Zoster vaccine reduced the incidence of zoster by 51.3% (63.9% in people 60 to 69 years old but only 37.6% in people at least 70 years old). The burden of disease was reduced by 61.1% (65.5% in people 60 to 69 years old and 55.4% in people at least 70 years old), and the duration of pain and discomfort among subjects with zoster was shorter in vaccinated candidates compared with placebo recipients. PHN was reduced by more than 65% for both age groups, and the most benefit was in the age group at least 70 years old. The study also showed that vaccination reduced the adverse impact of zoster on patients’ capacity to perform daily life activities and on health-related quality of life.

Zoster vaccine is safe. Rates of serious adverse events, systemic adverse events, hospitalizations, and deaths were low in vaccine recipients in the study and comparable to those in placebo recipients. During the first 42 days after vaccination, there were 24 cases of zoster in placebo recipients and 7 cases in the vaccination group, but none was caused by vOka. Unlike prophylactic vaccines such as those against varicella and measles, zoster vaccine is a therapeutic vaccine aimed at preventing reactivation of latent VZV in humans already infected before vaccination and thus with substantial immunity.

Zostavax can be given to patients with leukemia, to liver and gastrointestinal transplant recipients, and to patients with HIV infection, but is contraindicated in patients with bone marrow or lymphatic malignancy and patients receiving more than 20 mg/day of prednisone. Zoster vaccine was licensed by the US Food and Drug Administration (FDA) for healthy adults at least 50 years old to prevent zoster and its complications, mainly PHN. Post-licensure studies have confirmed the vaccine’s safety and efficacy. Unfortunately, participation in the zoster vaccine program has been low and this is likely due to cost and failure to recognize the importance of preventing infectious diseases in older adults. The Shingles Prevention Study demonstrated efficacy for 3 years after vaccination, whereas subsequent studies indicated efficacy for 8 years ^65^. Currently, the CDC does not recommend booster doses of zoster vaccine, but may in the future.

### GlaxoSmithKline vaccine

Recently, a liposome-based subunit vaccine, HZ/su, containing the VZV glycoprotein E and the adjuvant ASO1B was created. Studies revealed that two doses of HZ/su containing 50 µg of recombinant VZV glycoprotein E administered at 1- or 2-month intervals were well tolerated and induced much more robust VZV-specific and VZV glycoprotein E-specific CD4 ^+^ T-cell and antibody responses than did vOka ^66, 67^. A randomized placebo-controlled study of 15,411 subjects at least 50 years of age revealed a remarkable 97.2% efficacy in preventing zoster for a 3.2-year period that did not diminish with increasing age ^68^. Systemic adverse reactions were 2.2-fold greater in the vaccine group compared with the placebo group. Because HZ/su does not replicate, it will be safe for immunosuppressed patients. The duration of its effect is unknown. HZ/su vaccine is not yet FDA-approved.
